# Comparative dosimetric analysis of deep inspiration breath-hold versus free breathing radiotherapy in lung cancer: special emphasis on cardiac and subcardiac structure protection

**DOI:** 10.1016/j.tipsro.2025.100371

**Published:** 2025-12-15

**Authors:** Biney Pal Singh, Ahmed Ali Chughtai, Jennifer Stock, Philipp Bruners, Michael J. Eble, Ahmed Allam Mohamed

**Affiliations:** a Department of Radiation Oncology, Medical Faculty, RWTH Aachen University Hospital, Aachen, Germany; b Department of Diagnostic and Interventional Radiology, RWTH Aachen University Hospital, Aachen, Germany; c Center for Integrated Oncology Aachen, Bonn, Cologne and Duesseldorf (CIO ABCD), Aachen, Germany

**Keywords:** Deep inspiration breath-hold, Lung cancer, Cardiac substructures, Respiratory gating, Dose sparing

## Abstract

**Background:**

Radiation therapy (RT) plays a central role in the treatment of lung cancer. However, cardiac and pulmonary toxicities remain major concerns. Deep inspiration breath-hold (DIBH) has been shown to improve target stability and reduce organ-at-risk (OAR) exposure, but data in lung cancer, particularly regarding cardiac substructures, remain limited.

**Materials and methods:**

We retrospectively analysed 32 patients with lung cancer treated with RT at our institution between 2020 and 2021. Each patient underwent planning CTs in both DIBH and free breathing (FB), generating a total of 64 treatment plans using VMAT (60 Gy in 30 fractions). Contouring followed ESTRO ACROP guidelines, including detailed segmentation of cardiac chambers and coronary arteries. Dosimetric endpoints included Dmean, D2%, and VxGy for lung, heart, and subcardiac structures. Statistical comparisons between DIBH and FB plans were performed.

**Results:**

DIBH significantly increased lung volume and improved all lung dosimetric parameters, including Dmean (13.13 vs. 14.49 Gy, p < 0.001) and V20Gy (24.37 % vs. 27.28 %, p < 0.001). Cardiac sparing was evident, with lower heart D2% (39.91 vs. 41.67 Gy, p = 0.012) and V45Gy (2.23 % vs. 2.53 %, p = 0.005). Notably, significant reductions were observed in the LAD (Dmean: 5.59 vs. 6.57 Gy, p = 0.004), LCX, and LV. Subgroup analyses demonstrated consistent dosimetric advantages across tumor laterality and level 7 nodal involvement.

**Conclusions:**

DIBH offers substantial and consistent dosimetric benefits in lung cancer RT, reducing radiation burden to pulmonary, cardiac, and coronary structures. Given the known association between cardiac dose and long-term morbidity, these findings support the broader implementation of DIBH, especially in anatomically challenging cases involving subcarinal nodes or left-sided tumors.

## Highlights

• Cardiac dose burden correlates with adverse cardiac events in lung cancer patients.
• DIBH offers consistent dosimetric benefit in pulmonary and cardiac metrics.
• The extent of dosimetric change depends on tumor site and lymph node involvement.
• In complex cases (e.g. subcarinal lymph nodes) DIBH shows greater dosimetric benefit.
• Dose reduction in cardiac substructures might reduce the risk of toxicities.

## Introduction

With ongoing demographic shifts, namely population aging, growth, and rising socioeconomic disparities, the global incidence and morbidity of cancer continue to increase.[1] Among these, with an estimated 2.5 million cases, lung cancer is the most common tumour, and the most common tumour in terms of deaths, with 1.8 million, in 2022, showing a global increase in the last decade.[1,2] In this context, not only is a well-structured healthcare system essential, but also the optimized implementation of therapeutic modalities. For patients with locally advanced or inoperable lung cancer, radiotherapy (RT) plays a pivotal role, underscoring the need for continual evaluation and refinement of its application.

Over the past decades, advances such as intensity-modulated radiotherapy (IMRT), volumetric modulated arc therapy (VMAT), and stereotactic ablative radiotherapy (SABR) have significantly enhanced the precision and conformality of RT [3]. Nevertheless, one of the persistent challenges in thoracic irradiation lies in managing breathing-induced motion, which affects both target coverage and sparing of organs at risk (OARs). To mitigate these uncertainties, techniques such as deep inspiration breath-hold (DIBH) have been developed [4]. As early as the 1990 s, studies demonstrated the clinical feasibility of breath-hold strategies, showing that tumor immobilization is both tolerable and reproducible for patients, while also enabling improved tumor coverage and reduced lung dose [5,6]. Since then, a growing body of literature has confirmed the advantages of DIBH over free breathing (FB) in thoracic RT, including significant reductions in lung and cardiac doses, and associated improvements in clinical outcomes, most notably, reductions in rates of radiation pneumonitis and overall mortality [[7], [8], [9], [10]].

Due to its anatomical and functional centrality, the heart and its substructures are frequently exposed during lung RT, making them vulnerable to both acute and late toxicities. Multiple studies have identified dose–response relationships between irradiation of cardiac and subcardiac structures and subsequent cardiovascular events [[11], [12], [13], [14]]. These effects not only limit the potential for safe dose escalation in lung cancer but may also adversely impact overall survival through increased risks of ischemia, arrhythmias, and heart failure. The reported incidence of severe (grade ≥ 3) cardiac events following chemoradiotherapy ranges from 10 % to 33 % [11,12,15,16].

With improving survival outcomes in lung cancer, there is a growing imperative to assess pre-treatment cardiovascular risk better and to integrate strategies for effective cardiac sparing within RT planning [13,16]. Against this backdrop, the present study aims to investigate the potential benefits of DIBH in RT planning for patients with lung cancer, particularly in comparison to FB with respect to dosimetric exposure of subcardiac structures, including heart chambers and coronary arteries.

## Materials and methods

This retrospective study included 32 patients with lung cancer who received radiotherapy at our institution between January 1, 2020, and December 31, 2021. Each patient underwent planning computed tomography (P-CT) using both deep inspiration breath-hold (DIBH) and free-breathing four-dimensional CT (4D-CT) techniques, resulting in a total of 64P-CT scans.

**Imaging and treatment planning**

P-CTs were acquired using a 16-slice CT scanner (Brilliance CT Big Bore Oncology, Philips Medical Systems, Cleveland, OH, USA) with settings: 120 kV, 146 mAs, pitch of 0.813, and slice thickness of 3 mm. DIBH CT scans utilized Catalyst™ (CRAD, Uppsala, Sweden), an optical surface imaging system monitoring the sternal area movement.

Positron emission tomography (PET-CT) scans were available for selected patients and helped with contour delineation. Data were anonymized and imported into Pinnacle3 treatment planning software (version 14.0; Philips Healthcare, Amsterdam, Netherlands).

**Volume delineation**

Target volumes were delineated following ESTRO ACROP guidelines. Gross Tumor Volume (GTVp) was defined on lung window CT images; internal Gross Tumor Volume (iGTVp) was delineated for FB-CT using respiratory phases (0 %, 50 %, 90 %). A margin of 6–8 mm (depending on histology: 6 mm for squamous cell carcinoma, 8 mm for adenocarcinoma and small cell lung carcinoma) around GTV within lung tissue formed Clinical Target Volumes (CTVp/iCTVp). Enlarged or metabolically active lymph nodes on contrast-enhanced CT or PET-CT were delineated as nodal GTV (GTVn). Mediastinal lymph node stations were delineated as nodal CTV (CTVn), excluding uninvolved critical structures (heart, great vessels, trachea, bronchial tree, esophagus). The Planning Target Volume (PTV) was created by expanding the combined CTV (CTVp + CTVn) by 5 mm isotropically.

Lungs were delineated separately and then combined to form the total lung. Additionally, the heart with its four chambers −the left atrium (LA), right atrium (RA), left ventricle (LV), and right ventricle (RV) − was defined. The right and left coronary arteries (RCA and LCA), along with their main branches, namely the posterior descending artery (PDA), left anterior descending (LAD), and left circumflex artery (LCX), were outlined as subcardial structures based on delineation atlases from Duane F et al. [17].

Initial delineations were performed by two radiation oncology trainees (BS and AC) and then reviewed by two experienced radiation oncologists (ME and AAM). In cases of discrepancy, contours were revised through consensus with a radiologist (PB).

**Treatment planning and evaluation**

VMAT plans, consisting of two full coplanar arcs with 6 MV photon energy, were generated for both FB and DIBH CT scans, totaling 64 plans. Dose calculations used a collapsed cone algorithm with a 3 × 3 × 3 mm^3^ dose grid. The prescribed dose was 60 Gy delivered in 30 fractions. Treatment planning was optimized to ensure a uniform dose distribution across the PTV, aiming to deliver at least 95 % of the prescribed dose to 100 % of the CTV and more than 95 % of the PTV. Plan quality was evaluated using the following dose–volume metrics: D2% (approximate maximum dose to the target/OAR), D98% (approximate minimum dose to the target), Dmean (average dose to the target or OAR), Dx (dose received by x% of the target/OAR volume), and VxGy (percentage of volume receiving at least x Gy). Organ-at-risk constraints included: spinal canal D2% < 45 Gy, mean liver dose < 32 Gy, mean lung dose < 17.5 Gy with V20Gy < 20 %, and mean heart dose < 26 Gy.

Since the primary aim of this study was to examine the intrinsic dose reduction achieved with DIBH, no explicit dose constraints or tolerance thresholds for subcardiac structures were adhered to while treatment planning. Nevertheless, our findings are later discussed in the light of reported constraints – succinctly summarized by Walls G et. al. [16].

**Dosimetric analysis**

For each DIBH and FB plan a dose-volume histogram (DVH) was generated. From these various dosimetric and volumetric parameters were obtained:

• PTV volume, as well as D2%, D50% and D98% of the PTV
• VRI, i.e. the volume of PTV enclosed by certain – in our case the 95 % − isodose line

Using these parameters following indices were calculated:

• conformity index (CI) = VRI/PTV
• homogeneity index (HI) = (D2% − D98%)/IR * 100, where RI represents the reference isodose

In addition to that, for comparing analysis of DIBH and FB plans following dosimetric data was recorded:

**Table:** 

Lung, total:	Dmean (Gy), V20Gy (%), V40Gy (%)
Lung, ipsilateral and contralateral:	Dmean (Gy), V5Gy (%), V10Gy (%), V20 (%), V40 (%)
Heart:	D2% (Gy), V20 Gy (%), V45 Gy (%), V60 Gy (%), Dmean (Gy)
Cardiac chambers: • left atrium and ventricle • right atrium and ventricle	D2% (Gy), V10 Gy (%), V20 Gy (%), V30 Gy (%), V40 Gy (%), Dmean (Gy)
Coronary arteries: • left coronary artery • LAD • LCX • right coronary artery • PDA	D2% (Gy), V5 Gy (%), V10 Gy (%), V20 Gy (%), V30 Gy (%), V40 Gy (%), Dmean (Gy)

**Statistical analysis**

Collected data was further analyzed using Microsoft Excel (Microsoft Corporation, Redmond, Washington, USA) and Python (Version 3.13.2); graphs were generated with GraphPad Prism (GraphPad Software, Boston, Massachusetts, USA) and Excel.

First, the normal distribution of the data was assessed with the Shapiro-Wilk-Test, using a p-value threshold of 0.05. Datasets with a p-value > 0.05 were considered normally distributed and were compared using a parametric, paired *t*-test. If one of the datasets was classified as non-parametric, the Wilcoxon signed-rank test was applied. In both tests, a p-value < 0.05 indicated a significant difference between the compared data groups.

**Ethical statement/considerations:**

The study was approved by the local ethics committee of RWTH Aachen University (Approval No.: EK 25–232).

## Results

**Patient Characteristics**

The study cohort comprised 32 patients with a median age of 69 years. Histologically, patients presented predominantly with squamous cell carcinoma (n = 12), small cell lung carcinoma (n = 9), adenocarcinoma (n = 8), and mixed or large cell neuroendocrine carcinoma (n = 3). Detailed staging and tumor localization are summarized in Supplementary Table 1.

**Target volume and dosimetric indices**

The mean PTV was slightly reduced in DIBH; 496.24 cm^3^ compared to FB; 511.32 cm^3^, though this difference did not reach statistical significance (p = 0.061). Both CI and HI demonstrated comparable results between techniques (CI: DIBH = 1.01, FB = 1.02, p = 0.36; HI: DIBH = 9.23, FB = 8.68, p = 0.09) (Table 1).

**Table 1:** Volumetric and dosimetric characteristics of investigated radiation plans in deep inspiration breath-hold (DIBH) and free breathing (FB).

**Volumetric parameter/dosimetric index**	**FB**	**DIBH**	**Test**	**p-value**
PTV volume	511.32 cm^3^	496.24 cm^2^	a	0.061
CI	1.02	1.01	a	0.36
HI	8.68	9.23	a	0.09
MU	656.23	695.79	a	0.12

**Lung dosimetry**

Relative to FB 4D-CT, DIBH scan produced markedly larger lung volumes, expanding the whole lung by an average of 1,602.7 cm^3^, the ipsilateral lung by 722.11 cm^3^, and the contralateral lung by 873.25 cm^3^.

**Fig. 1:**
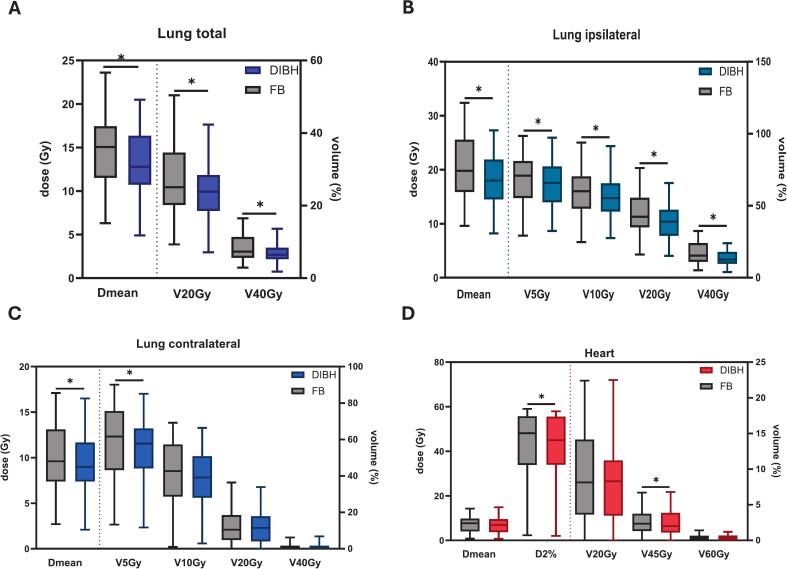
Boxplots showing dosimetric outcome of lung total (A), lung ipsilateral (B), lung contralateral (C) and heart (D) comparing results of DIBH plan (colourful) and FB plan (grey).

This volumetric gain translated into clear dosimetric advantages: the lung Dmean fell from 14.49 Gy with FB to 13.13 Gy with DIBH (p < 0.001), as well as in volume parameters V20Gy (24.37 % vs. 27.28 %, p < 0.001) and V40Gy (6.67 % vs. 8.40 %, p < 0.001) (Fig. 1, 2Table 2).

Focusing on the ipsilateral lung, DIBH consistently reduced every evaluated parameter, including Dmean (18.11 vs. 20.46 Gy, p < 0.001), V5Gy, V10Gy, V20Gy, and V40Gy. The contralateral lung demonstrated significant improvements, primarily in Dmean (9.08 vs. 9.62 Gy, p = 0.009) and V5Gy (54.57 % vs. 58.28 %, p = 0.001) (Fig. 1, Table 2).

**Table 2:** All significantly different dosimetric parameters in planning in deep inspiration breath-hold (DIBH) and free breathing (FB), a = paired *t*-test, b = Wilcoxon signed-rank test.

	**FB**	**DIBH**		**Test**	**p-value**
*Lung total*					
Dmean (Gy)	14.49	13.13	−9.39	a	**2.59365E-05**
V20Gy (%)	27.28	24.37	−10.67	a	**0.000430897**
V40Gy / %	8.4	6.67	−20.60	a	**1.27E-05**
*Lung ipsilateral*					
Dmean / Gy	20.46	18.11	−11.49	a	**8.28E-06**
V5Gy / %	69.47	65.27	−6.05	a	**8.65E-05**
V10Gy / %	60.36	55.79	−7.57	a	**9.56E-05**
V20Gy / %	44.77	39.43	−11.93	a	**4.04E-05**
V40Gy / %	17.23	13.53	−21.47	a	**3.85E-06**
*Lung contralateral*					
Dmean / Gy	9.62	9.08	−5.61	a	**0.009350664**
V5Gy / %	58.28	54.57	−6.37	a	**0.001208776**
*Heart*					
D2% / Gy	41.67	39.91	−4.22	b	**0.012457265**
V45 Gy / %	2.53	2.23	−11.86	b	**0.00548476**
*Left ventricle*					
D2% / Gy	12.38	10.53	−14.94	b	**0.00968003**
Dmean / Gy	4.15	3.76	−9.40	b	**0.008160867**
*Right atrium*					
D2% / Gy	26.79	23.84	−11.01	b	**0.031105267**
V30 Gy / %	4.68	3.27	−30.13	b	**0.012407092**
V40 Gy / %	2.13	1.39	−34.74	b	**0.000843013**
*Left coronary artery*					
D2% / Gy	15.23	13.05	−14.31	b	**0.020216676**
*Left circumflex coronary*					
V5 Gy / %	49.33	43.55	−11.72	b	**0.002699796**
Dmean / Gy	9.6	8.85	−7.81	b	**0.014803257**
*Left anterior descending*					
D2% / Gy	15.21	12.32	−19.00	b	**0.00154797**
V5 Gy / %	49.68	39.39	−20.71	b	**0.001435352**
Dmean / Gy	6.57	5.59	−14.92	b	**0.00438754**
*Posterior descending artery*					
D2% / Gy	2.2	2.08	−5.45	b	**0.044224421**
Dmean / Gy	1.63	1.6	−1.84	b	**0.026056039**

**Cardiac and subcardiac structure dosimetry**

The DIBH approach resulted in a significant reduction in heart volume (516.4 vs 547.3 cm^3^, p < 0.001). Additionally, significant dosimetric improvements were observed in the heart's D2% (39.91 Gy in DIBH vs. 41.67 Gy in FB, p = 0.012) and V45 Gy (2.23 % in DIBH vs. 2.53 % in FB, p = 0.005). Heart Dmean and V20Gy showed reductions in DIBH but did not reach statistical significance (p = 0.078 and p = 0.110, respectively) (Fig. 1, Table 2). No significant difference was observed for V60G.

**Fig. 2:**
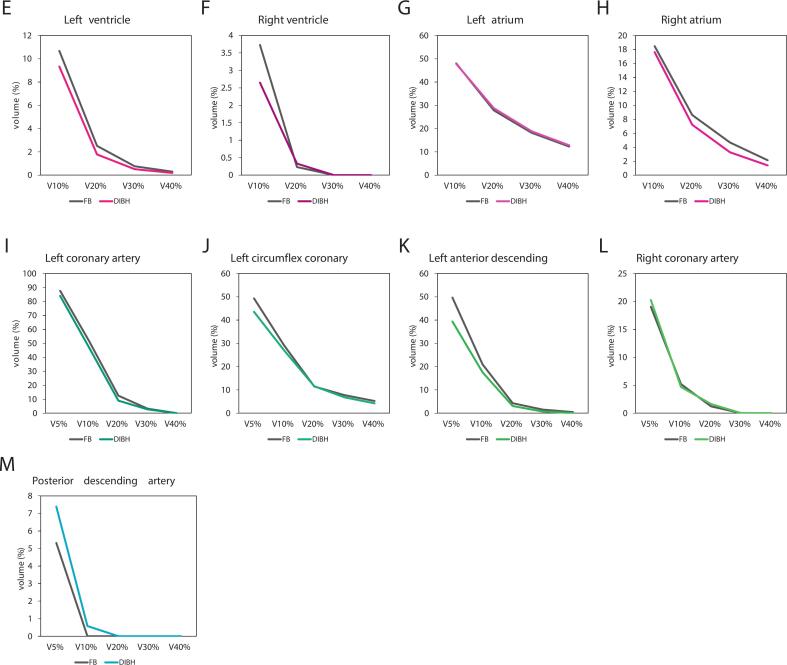
Linear diagrams showing relative volumes in relation to dose for the left ventricle (E), right ventricle (F), left atrium (G), right atrium (H), left coronary artery (I), left circumflex coronary (J), left anterior descending (K), right coronary artery (L), posterior descending artery (M).

Subcardiac analysis showed that DIBH significantly reduced radiation exposure to the LV, with decreases in D2% (10.53 vs. 12.38 Gy, p = 0.009) and Dmean (3.76 vs. 4.15 Gy, p = 0.008). No significant dosimetric differences were found for the RV (Fig. 2, Table 2).

For atrial structures, significant dose reductions with DIBH were limited to the RA, including D2% (23.84 vs. 26.79 Gy, p = 0.031), V30Gy (3.27 % vs. 4.68 %, p = 0.012), and V40Gy (1.39 % vs. 2.13 %, p < 0.001).

In the coronary arteries, DIBH significantly reduced D2% for the LCA (13.05 vs. 15.23 Gy, p = 0.020), LAD (12.32 vs. 15.21 Gy, p = 0.002), and PDA (2.08 vs. 2.20 Gy, p = 0.044). Additionally, Dmean was significantly lower for LAD (5.59 vs. 6.57 Gy, p = 0.004), PDA (1.60 vs. 1.63 Gy, p = 0.026), and LCX (8.85 vs. 9.60 Gy, p = 0.015). No significant differences were observed for the RCA (Fig. 2, Table 2).

**Subgroup analyses**

A subgroup analysis was performed to assess the effect of tumor laterality (left versus right lung tumors) and the presence or absence of subcarinal lymph node metastases (thoracic level station seven, “LS 7) on the observed dosimetric advantages of DIBH. Laterality of tumors was evenly distributed – 17 patients had tumors of the left and 15 patients of the right lung. While 22 patients demonstrated involvement of lymph node station 7, it was absent in 10.

For tumors of the left lung, DIBH significantly enhanced pulmonary and cardiac dosimetry. For the total lung, Dmean decreased from 14.04 Gy to 12.86 Gy (p = 0.002), with notable reductions in V20Gy (25.72 % to 23.72 %, p = 0.032) and V40Gy (7.60 % to 6.31 %, p = 0.002). Ipsilateral lung metrics consistently showed significant improvements, with decreases in Dmean (from 20.20 Gy to 17.94 Gy, p < 0.001) and V40Gy (from 16.56 % to 13.23 %, p < 0.001). For the contralateral lung, both Dmean (9.88 Gy to 9.30 Gy, p = 0.042) and V5Gy (58.89 % to 54.60 %, p = 0.006) significantly declined. Cardiac exposure was significantly reduced in both the LCX (D2%: 29.83 Gy to 25.46 Gy, p = 0.005; Dmean: 13.48 Gy to 11.99 Gy, p = 0.008) and LAD (D2%: 18.66 Gy to 14.41 Gy, p = 0.015; Dmean: 8.34 Gy to 7.09 Gy, p = 0.043) (Table 3).

**Table 3:** All significantly different dosimetric parameters in planning in deep inspiration breath-hold (DIBH) and free breathing (FB) of patients with left or right lungs tumours, a = paired *t*-test, b = Wilcoxon signed-rank test.

**Tumor in the LEFT Lung**	**FB**	**DIBH**		**Test**	**p-value**
*Lung total*					
Dmean (Gy)	14.04	12.86	−8.40	a	**0.001714318**
V20Gy (%)	25.72	23.72	−7.78	a	**0.031620242**
V40Gy / %	7.6	6.31	−16.97	a	**0.002472702**
*Lung ipsilateral*					
Dmean / Gy	20.2	17.94	−11.19	a	**0.000261375**
V5Gy / %	70.38	65.35	−7.15	a	**6.25577E-05**
V10Gy / %	60.21	55.38	−8.02	a	**0.000407702**
V20Gy / %	43.61	39.11	−10.32	a	**0.004194853**
V40Gy / %	16.56	13.23	−20.11	a	**0.000332789**
*Lung contralateral*					
Dmean / Gy	9.88	9.3	−5.87	a	**0.042332474**
V5Gy / %	58.89	54.6	−7.28	a	**0.006134563**
*Left circumflex coronary*					
D2% / Gy	29.83	25.46	−14.65	b	**0.004638672**
V5 Gy / %	56.32	50.19	−10.88	b	**0.009925486**
Dmean / Gy	13.48	11.99	−11.05	b	**0.007904053**
*Left anterior descending*					
D2% / Gy	18.66	14.41	–22.78	a	**0.015299659**
V5 Gy / %	58.46	50.81	−13.09	a	**0.017054667**
Dmean / Gy	8.34	7.09	−14.99	a	**0.042789071**
**Tumor in the RIGHT Lung**					
*Lung total*					
Dmean (Gy)	15	13.44	−10.40	a	**0.005285044**
V20Gy (%)	29.05	25.11	−13.56	a	**0.006200701**
V40Gy / %	9.31	7.09	–23.85	a	**1.59E-03**
*Lung ipsilateral*					
Dmean / Gy	20.75	18.3	−11.81	a	**7.35E-03**
V10Gy / %	60.53	56.25	−7.07	a	**3.55E-02**
V20Gy / %	46.07	39.8	−13.61	a	**4.30E-03**
V40Gy / %	18	13.86	–23.00	b	**6.10E-04**
*Heart*					
V45 Gy / %	2.83	2.47	−12.72	a	**0.008692475**
Dmean / Gy	7.17	6.53	−8.93	a	**0.034038065**
*Left ventricle*					
Dmean / Gy	2.42	2.23	−7.85	b	**0.012451172**
*Right atrium*					
D2% / Gy	39.85	32.89	−17.47	a	**0.005337672**
V20 Gy / %	16.79	12.99	–22.63	b	**0.033046944**
V30 Gy / %	9.56	6.49	–32.11	b	**0.003701749**
V40 Gy / %	4.51	2.93	−35.03	b	**0.00146885**
Dmean / Gy	10.09	8.55	−15.26	b	**0.008361816**
*Left anterior descending*					
V5 Gy / %	39.73	26.45	–33.43	a	**0.020891279**

For right-lung tumors, significant dosimetric benefits were observed in the total lung, including reductions in Dmean (from 15 Gy to 13.44 Gy, p = 0.005), V20Gy (from 29.05 % to 25.11 %, p = 0.006), and V40Gy (from 9.31 % to 7.09 %, p = 0.002). The ipsilateral lung showed significant decreases in Dmean (20.75 Gy to 18.30 Gy, p = 0.007), V20Gy (46.07 % to 39.80 %, p = 0.004), and V40Gy (18.00 % to 13.86 %, p < 0.001). Cardiac sparing was also prominent, as demonstrated by reductions in heart V45Gy (2.83 % to 2.47 %, p = 0.009), LV Dmean (2.42 Gy to 2.23 Gy, p = 0.012), and substantial decreases in RA- dose metrics, particularly D2% (39.85 Gy to 32.89 Gy, p = 0.005) and V40Gy (4.51 % to 2.93 %, p = 0.001). Additionally, V5Gy of LAD decreased from 39.73 % to 26.45 % (p = 0.021) (Table 3).

Further, patients with positive subcarinal lymph nodes demonstrated particularly pronounced dosimetric advantages with DIBH. Cardiac dosimetry showed improvements, including heart V45Gy (3.08 % to 2.72 %, p = 0.012), and significant reductions in LV D2% (13.51 Gy to 11.01 Gy, p = 0.017). Coronary artery exposure was also reduced, particularly in LAD D2% (16.30 Gy to 13.67 Gy, p = 0.007), Dmean (7.14 Gy to 6.09 Gy, p = 0.016), and PDA's Dmean (1.87 Gy to 1.87 Gy, p = 0.040) (Table 4).

**Table 4:** All significantly different dosimetric parameters in planning in deep inspiration breath-hold (DIBH) and free breathing (FB) of patients with or without involvement of lymph nodes station 7, a = paired *t*-test, b = Wilcoxon signed-rank test.

**LS 7 positive**	**FB**	**DIBH**		**Test**	p-value
*Lung total*					
Dmean (Gy)	16.11	14.52	−9.87	a	**0.00020377**
V20Gy (%)	31.13	27.62	−11.28	a	**0.001696284**
V40Gy / %	8.77	6.94	−20.87	a	**2.98E-04**
*Lung ipsilateral*					
Dmean / Gy	21.27	18.89	−11.19	a	**1.95E-04**
V5Gy / %	73.92	69.72	−5.68	a	**7.30E-04**
V10Gy / %	64.44	60.16	−6.64	a	**1.95E-03**
V20Gy / %	46.77	41.28	−11.74	a	**9.25E-04**
V40Gy / %	17.06	13.36	−21.69	a	**4.77E-07**
*Lung contralateral*					
Dmean / Gy	11.64	10.83	−6.96	a	**0.003460223**
V5Gy / %	68.14	62.86	−7.75	a	**0.000397692**
V10Gy / %	49.53	46.47	−6.18	a	**0.018867291**
*Heart*					
V45 Gy / %	3.08	2.72	−11.69	b	**0.01163652**
*Left ventricle*					
D2% / Gy	13.51	11.01	−18.50	b	**0.017245169**
Dmean / Gy	4.42	3.94	−10.86	b	**0.036495955**
*Right atrium*					
V40 Gy / %	2.22	1.54	−30.63	b	**0.003494192**
*Left circumflex coronary*					
V5 Gy / %	56.94	51.21	−10.06	b	**0.02490692**
Dmean / Gy	10.51	9.75	−7.23	b	**0.03587532**
*Left anterior descending*					
D2% / Gy	16.3	13.67	−16.13	b	**0.007443428**
V5 Gy / %	54.3	44.05	−18.88	a	**0.005592619**
Dmean / Gy	7.14	6.09	−14.71	b	**0.015698997**
*Posterior descending artery*					
Dmean / Gy	1.87	1.87	0.00	b	**0.039833215**
					
**LS 7 negative**					
*Lung total*					
V40Gy / %	7.59	6.09	−19.76	a	**2.23E-02**
*Lung ipsilateral*					
Dmean / Gy	18.66	16.38	−12.22	a	**2.38E-02**
V10Gy / %	51.39	46.16	−10.18	a	**2.71E-02**
V20Gy / %	40.35	35.38	−12.32	a	**2.21E-02**
V40Gy / %	17.63	13.89	−21.21	a	**2.08E-02**
*Left coronary artery*					
D2% / Gy	11.12	9.61	−13.58	a	**0.014399122**
*Left circumflex coronary*					
V5 Gy / %	32.59	26.7	−18.07	b	**0.027707849**

In patients without subcarinal lymph node involvement, significant improvements in coronary artery exposure were limited to the LCA D2% (11.12 Gy to 9.61 Gy, p = 0.014) and LCX V5Gy (32.59 % to 26.70 %, p = 0.028) (Table 4).

Overall, across all evaluated subgroups, DIBH consistently maintained or enhanced the favorable dosimetric profile compared to FB, without statistically significant deterioration of any evaluated parameter.

## Discussion

The pivotal role of radiotherapy in lung cancer management is well-established, as is its association with potential adverse cardiac effects. Evidence from recent studies has emphasized that radiation exposure to cardiac structures, particularly the coronary arteries and cardiac chambers, significantly increases the risk of major adverse cardiac events (MACE), arrhythmias, and mortality [18,19].

Atkins et al. highlighted that an increased mean cardiac dose and LAD V15Gy ≥ 10 % are independent predictors of MACE and all-cause mortality. [18] Similar findings were reinforced by systematic reviews, underscoring the necessity of dose optimization to cardiac and subcardiac structures to mitigate cardiovascular morbidity and mortality following RT for lung cancer [14,16].

Beyond immediate dosimetric considerations, the long-term implications of cardiac exposure during thoracic RT have gained increasing attention. Accelerated coronary artery disease, often termed RICAD, is now recognized as a distinct clinical entity, often affecting proximal coronary segments such as the LAD and LCX, with a latency period that may extend beyond a decade post-RT. As highlighted by Kirresh et al., even moderate doses to these vessels can accelerate atherosclerotic changes, leading to clinically significant coronary events, particularly in patients with pre-existing cardiovascular risk factors.[20] Importantly, RICAD is frequently underdiagnosed due to its overlap with traditional ischemic heart disease and the lack of routine cardiac surveillance in long-term cancer survivors.

Also, Wiedemann et al. showed that thoracic radiotherapy is associated with remodelling processes of the pulmonary vessels, which can be associated with cardiac diseases.[21] The incidences of severe cardiac events, i.e. grade 3 and higher, in patients with lung cancer after chemoradiotherapy range from 10 to 33 %, depending on the observed study [11,12,15,16], making prudent radiotherapy planning with consistent protection of the pulmonary and cardiac OARs essential. The use of DIBH can contribute to that goal.

The positive effects of DIBH use on the dosimetric outcome for pulmonary, cardiac, and subcardiac structures have been recorded in various studies. General effects have been described in all thoracic malignancies. Benefits for cardiac substructures are most particularly demonstrated in breast cancer patients [[22], [23], [24], [25], [26]]. A comparative study for a group of oesophageal cancer patients was conducted, emphasising the importance of DIBH for dose reduction in cardiac substructures [27]. To our knowledge, our now presented work is the first to give a detailed overview of dosimetric changes in cardiac substructures in DIBH compared to FB in lung cancer.

Analysing the entire group, a significant improvement was observed in all dosimetric parameters of the total and ipsilateral lung. Additionally, parameters of the contralateral lung improved with DIBH, but only Dmean and V5Gy showed significant changes. The underlying mechanisms, namely, an increase in lung volume and a reduction of target volume mobility and lung tissue density, have been described, and their benefits have been repeatedly demonstrated [[5], [6], [7],^9^,^10^,^28^].

The observed cardiac sparing effects, notably significant reductions in heart’s D2% and V45Gy, echo the results of previously published literature, which reported improved dosimetric outcomes with the implementation of DIBH [29]. Recent Systematic reviews have consistently highlighted a dose–response relationship between cardiac irradiation and subsequent cardiovascular events, reinforcing the importance of precise cardiac dose management [14,16].

In evaluating specific cardiac substructures, we observed significant dosimetric improvements in the LV and RA, results that should be considered alongside the findings by Jang et al., who identified the LV as especially sensitive to radiation dose and strongly linked to increased cardiac event risks [12]. Our findings on coronary artery sparing, particularly the LAD, LCX, and PDA, provide important evidence supporting the adoption of DIBH in clinical practice as possible to potentially reduce ischemic cardiac events [30,31].

A notable finding in our analysis was the varied impact of DIBH based on tumor laterality. Patients with left-sided tumors showed significant reductions in coronary artery doses. Specifically, V5Gy, D2%, and Dmean of the LCX and LAD were significantly lowered by DIBH, highlighting the benefit of sparing the LAD in reducing cardiac events after RT, which is well documented in breast cancer. However, unlike the entire cohort, there was no significant dose reduction in heart parameters overall or specifically in the left ventricle. The most noteworthy improvement in the group with right lung tumors was observed in parameters related to the right atrium. Patients experienced dose reductions across nearly all parameters, including V5 Gy of the LAD. The varying degrees of dose reduction can be explained by the different radiation exposure of the respective structures and the movement and deformation of the heart during DIBH. The LCX and LAD which due to their left-sided location are more affected while irradiation of same-sided tumors they experience a dose improvement because of the compression of the heart and the shift of caudal parts of heart from the left to the center. In contrast, the burden on the right atrium is considerably higher in radiation of right-sided carcinomas. Therefore, the narrowing of the heart while DIBH shows more pronounced dose sparing in these tumors [8].

Given these findings, we further explored whether the benefit of DIBH is maintained in anatomically complex cases, specifically when RT fields include LS 7 due to nodal involvement in the inferior mediastinum and proximity to the heart, we specifically analysed the impact of DIBH on RT dose distribution in relation to whether this nodal station required inclusion in the target volume. In patients with LS 7-positive disease, DIBH significantly reduced radiation exposure to both pulmonary and cardiac OARs compared to FB. Lung sparing was consistent across all evaluated parameters, including total lung Dmean, V20, and V40, as well as ipsilateral and contralateral lung sub-volumes, indicating that the benefits of DIBH are maintained even when target volumes extend to the inferior mediastinum. Moreover, cardiac dose was also significantly reduced, with lower V45, Dmean and D2% of the LV, and V5/Dmean of both the LCX and LAD, underscoring the cardioprotective potential of DIBH in this setting. In general, it should be noted that in our cohort the baseline dose exposure of pulmonary and cardiac structures is lower in LS 7-negative carcinomas. Previous studies have already shown, for example, significantly higher cardiac doses due to multistation nodal involvement [32]. With considerably higher exposure in subcarinal involvement, there is greater potential for dose reduction in this subgroup when DIBH is used.

The overall cardioprotective potential is underlined even more when above mentioned findings are placed in context of published dose relationships and thresholds for cardiac events, for example compiled by Walls G et al. [16] Among others the Dmean of both ventricles and the LAD is listed as a parameter correlating with ischemia and heart failure in radiated patients with lung cancer. DIBH usage reduced – even though not significantly – the Dmean of the right ventricle in the total, the right tumor cohort, and both LS 7 subgroups. Same metric was bettered significantly for the left ventricle in total cohort, for right-sided and LS 7 positive tumors. LAD’s Dmean was improved significantly in the complete cohort, and the left tumour and LS 7 positive subgroups. The V30 of the left ventricle is also associated with the development of ischemia; DIBH decreased the value in the total cohort, and in left sided and LS 7 positive tumors.

Since neither all volumetric and dosimetric values were assessed, nor all structures were contoured, our data cannot completely be positioned within existing explicit thresholds and constraints for all adverse cardiac events. Nevertheless, the parameters which are collected already emphasize the benefit of DIBH for patients’ clinical outcome after lung cancer therapy in the light of published literature.

Despite these promising findings, the implementation of DIBH in clinical practice is not without challenges. Effective DIBH requires consistent patient cooperation and breath-hold reproducibility, which can be difficult for individuals with compromised pulmonary function or limited compliance. Thus, although all patients in our cohort were initially able to perform DIBH-CT, only eleven patients’ radiation plans were ultimately planned on those. Motion management technologies such as surface-guided RT or respiratory gating systems are often necessary to monitor breath-hold accuracy but add technical complexity and require additional resources. Moreover, integrating DIBH into routine clinical workflows demands dedicated staff training and patient preparation, which may impact efficiency in high-volume centers [33,34]. These considerations must be weighed when evaluating the feasibility and scalability of DIBH in broader clinical practice.

## Study limitations

Our study has several limitations that should be acknowledged. Firstly, the retrospective design and relatively small sample size may limit the generalizability of our findings and the statistical power to detect subtle differences. Secondly, the study lacks clinical follow-up data to correlate dosimetric advantages with actual clinical outcomes such as reduced cardiac and pulmonary toxicity. Additionally, interobserver variability in contour delineation, despite standardized guidelines and consensus reviews, could have introduced minor inconsistencies. Furthermore, patient compliance and reproducibility of the DIBH technique could vary, potentially affecting dosimetric accuracy. Lastly, due to the retrospective nature of the study, potential confounding variables such as patient comorbidities and baseline cardiac function were not fully controlled, necessitating future prospective studies to validate our results comprehensively.

## Conclusion

Overall, our study robustly supports the implementation of DIBH in lung cancer radiotherapy planning, demonstrating dosimetric benefits in the majority of cardiac and pulmonary parameters, and in a multitude of these even significant improvements. Aligning with recommendations from recent systematic reviews, our results reinforce the importance of individualized radiation planning, meticulous cardiac structure delineation, and proactive dose-sparing strategies to optimize therapeutic outcomes and patient quality of life.

**Waiver of patient consent**

This is a retrospective case study. Patient consent has been waived by Ethic committee.

**Author Contributions:**

Manuscript drafting and editing (BPS), revision of Manuscript (AMM, MJE), Conception and design of the study (BPS, AAC, MJE), Segmentation of target volumes and OAR (BPS, AAC), Revision of the segmentation (PB, AAM, MJE), Radiation treatment planning (JS) Data collection (BPS), statistical analysis (BPS, AAM), Revision of the analysis (AAM, MJE).

## Funding

This research received no specific grant from funding agencies in the public, commercial, or not-for-profit sectors.

## Declaration of competing interest

The authors declare that they have no known competing financial interests or personal relationships that could have appeared to influence the work reported in this paper.
