# Supplementary material for: Comparative dosimetric analysis of deep inspiration breath-hold versus free breathing radiotherapy in lung cancer: special emphasis on cardiac and subcardiac structure protection
Source: Tech Innov Patient Support Radiat Oncol. 2025 Dec 15;37:100371. doi: 10.1016/j.tipsro.2025.100371 (PMC12774762; doi:10.1016/j.tipsro.2025.100371)
Supplement: Supplementary Data 1 [file mmc1.docx]

| Patient number | Age | Sex | Histology | Tumour stage | Tumour localisation |
| --- | --- | --- | --- | --- | --- |
| 1 | 64 | m | adenocarcinoma | cT4, N2 | right upper lobe |
| 2 | 73 | f | squamous cell carcinoma | cT3, N0 | right upper lobe |
| 3 | 78 | m | squamous cell carcinoma | cT4, N2 | left lower lobe |
| 4 | 78 | m | squamous cell carcinoma | cT3, N2 | left lower lobe |
| 5 | 58 | m | small cell lung carcinoma | cT1b, N2 | left upper lobe |
| 6 | 65 | f | adenocarcinoma | cT2b, N2 | right upper lobe |
| 7 | 76 | m | adenocarcinoma | cT3, N3 | left lower lobe |
| 8 | 75 | m | squamous cell carcinoma | cT3, N1 | left upper lobe |
| 9 | 69 | f | small cell lung carcinoma | cT3, N2 | right lower lobe |
| 10 | 79 | m | squamous cell carcinoma | cT3, N1 | left lower lobe |
| 11 | 53 | m | adenocarcinoma | cT4, N3 | left lower lobe |
| 12 | 68 | f | small cell lung carcinoma | cT4, N3 | left upper lobe |
| 13 | 68 | f | squamous cell carcinoma | cT3, N0 | right lower lobe |
| 14 | 79 | m | small cell lung carcinoma | cT3, N3 | right upper lobe |
| 15 | 71 | f | adenocarcinoma | cT1c, N3 | left upper lobe |
| 16 | 74 | m | squamous cell carcinoma | cT2a, N3 | lingula |
| 17 | 59 | f | small cell lung carcinoma | cT2, N3 | right upper lobe |
| 18 | 69 | m | squamous cell carcinoma | cT2a, N1 | right upper lobe |
| 19 | 56 | f | adenocarcinoma | cT1c, N3 | right upper lobe |
| 20 | 79 | m | squamous cell carcinoma | cT3, N3 | left upper lobe |
| 21 | 64 | m | squamous cell carcinoma | cT4, N3 | left upper lobe |
| 22 | 55 | m | large cell neuroendocrine carcinoma | cT4, N2 | right lung, beyond lobes |
| 23 | 85 | m | squamous cell carcinoma | cT3, N2 | right middle lobe |
| 24 | 54 | m | large cell neuroendocrine carcinoma | cT1a, N3 | right upper lobe |
| 25 | 72 | F | small cell lung carcinoma | cT3, N1 | left lower lobe |
| 26 | 86 | m | small cell lung carcinoma | cT4, N2 | right upper lobe |
| 27 | 63 | m | adenocarcinoma | cT1b, cN2 | left upper lobe |
| 28 | 62 | m | small cell lung carcinoma | cT3, N2 | right lung, beyond lobes |
| 29 | 64 | m | small cell lung carcinoma | cT2a, N3 | left upper lobe |
| 30 | 71 | m | adenocarcinoma | cT2a, N2 | left lower lobe |
| 31 | 66 | m | squamous cell carcinoma | cT2a, N2 | right upper lobe |
| 32 | 80 | f | large cell neuroendocrine carcinoma | cT4, N0 | left lower lobe |

Supplementary Table 1: Patients characteristics.

|  | **FB** | **DIBH** | **Mean relative  difference (%)** | **Test** | **p-value** |
| --- | --- | --- | --- | --- | --- |
| *Lung total* |  |  |  |  |  |
| Dmean (Gy) | 14.49 | 13.13 | -9.39 | a | **2.59365E-05** |
| V20Gy (%) | 27.28 | 24.37 | -10.67 | a | **0.000430897** |
| V40Gy / % | 8.4 | 6.67 | -20.60 | a | **1.27E-05** |
| *Lung ipsilateral* |  |  |  |  |  |
| Dmean / Gy | 20.46 | 18.11 | -11.49 | a | **8.28E-06** |
| V5Gy / % | 69.47 | 65.27 | -6.05 | a | **8.65E-05** |
| V10Gy / % | 60.36 | 55.79 | -7.57 | a | **9.56E-05** |
| V20Gy / % | 44.77 | 39.43 | -11.93 | a | **4.04E-05** |
| V40Gy / % | 17.23 | 13.53 | -21.47 | a | **3.85E-06** |
| *Lung contralateral* |  |  |  |  |  |
| Dmean / Gy | 9.62 | 9.08 | -5.61 | a | **0.009350664** |
| V5Gy / % | 58.28 | 54.57 | -6.37 | a | **0.001208776** |
| V10Gy / % | 39.27 | 37.72 | -3.95 | a | 0.125608751 |
| V20Gy / % | 12.98 | 12.01 | -7.47 | b | 0.643191592 |
| V40Gy / % | 1.18 | 1.16 | -1.69 | b | 0.887979046 |
| *Heart* |  |  |  |  |  |
| D2% / Gy | 41.67 | 39.91 | -4.22 | b | **0.012457265** |
| V20 Gy / % | 9.07 | 8.15 | -10.14 | a | 0.110277863 |
| V45 Gy / % | 2.53 | 2.23 | -11.86 | b | **0.00548476** |
| V60 Gy/ % | 0.4 | 0.38 | -5.00 | b | 0.696346664 |
| Dmean / Gy | 7.27 | 6.91 | -4.95 | a | 0.077763842 |
| *Left ventricle* |  |  |  |  |  |
| D2% / Gy | 12.38 | 10.53 | -14.94 | b | **0.00968003** |
| V10 Gy / % | 10.68 | 9.33 | -12.64 | b | 0.395696234 |
| V20 Gy / % | 2.51 | 1.78 | -29.08 | b | 0.202328308 |
| V30 Gy / % | 0.76 | 0.51 | -32.89 | b | 0.32698935 |
| V40 Gy / % | 0.3 | 0.19 | -36.67 | b | 0.345231072 |
| Dmean / Gy | 4.15 | 3.76 | -9.40 | b | **0.008160867** |
| *Right ventricle* |  |  |  |  |  |
| D2% / Gy | 7.44 | 6.68 | -10.22 | b | 0.268159443 |
| V10 Gy / % | 3.73 | 2.65 | -28.95 | b | 0.57598703 |
| V20 Gy / % | 0.23 | 0.33 | 43.48 | b | 1 |
| V30 Gy / % | 0 | 0 | 0.00 |  |  |
| V40 Gy / % | 0 | 0 | 0.00 |  |  |
| Dmean / Gy | 2.88 | 2.69 | -6.60 | b | 0.245303636 |
| *Left atrium* |  |  |  |  |  |
| D2% / Gy | 48.22 | 47.44 | -1.62 | b | 0.295070265 |
| V10 Gy / % | 48.03 | 47.81 | -0.46 | b | 0.991794348 |
| V20 Gy / % | 27.81 | 28.76 | 3.42 | a | 0.560752212 |
| V30 Gy / % | 18.21 | 18.86 | 3.57 | a | 0.585957439 |
| V40 Gy / % | 12.26 | 12.8 | 4.40 | b | 0.478455666 |
| Dmean / Gy | 15.94 | 16.1 | 1.00 | a | 0.794055103 |
| *Right atrium* |  |  |  |  |  |
| D2% / Gy | 26.79 | 23.84 | -11.01 | b | **0.031105267** |
| V10 Gy / % | 18.47 | 17.62 | -4.60 | b | 0.374043408 |
| V20 Gy / % | 8.6 | 7.21 | -16.16 | b | 0.326027571 |
| V30 Gy / % | 4.68 | 3.27 | -30.13 | b | **0.012407092** |
| V40 Gy / % | 2.13 | 1.39 | -34.74 | b | **0.000843013** |
| Dmean / Gy | 7.18 | 6.58 | -8.36 | b | 0.112336656 |
| *Left coronary artery* |  |  |  |  |  |
| D2% / Gy | 15.23 | 13.05 | -14.31 | b | **0.020216676** |
| V5 Gy / % | 87.66 | 84.01 | -4.16 | b | 0.172954918 |
| V10 Gy / % | 51.45 | 46.8 | -9.04 | b | 0.454888845 |
| V20 Gy / % | 12.53 | 8.98 | -28.33 | b | 0.423596318 |
| V30 Gy / % | 3.23 | 2.72 | -15.79 | b | 0.179712495 |
| V40 Gy / % | 0 | 0 |  |  |  |
| Dmean / Gy | 11.63 | 10.51 | -9.63 | b | 0.06784779 |
| *Left circumflex coronary* |  |  |  |  |  |
| D2% / Gy | 21.09 | 19.43 | -7.87 | b | 0.09773239 |
| V5 Gy / % | 49.33 | 43.55 | -11.72 | b | **0.002699796** |
| V10 Gy / % | 29.1 | 26.96 | -7.35 | b | 0.469162525 |
| V20 Gy / % | 11.48 | 11.58 | 0.87 | b | 0.876701302 |
| V30 Gy / % | 7.75 | 6.83 | -11.87 | b | 0.498962299 |
| V40 Gy / % | 5.23 | 4.22 | -19.31 | b | 0.128190174 |
| Dmean / Gy | 9.6 | 8.85 | -7.81 | b | **0.014803257** |
| *Left anterior descending* |  |  |  |  |  |
| D2% / Gy | 15.21 | 12.32 | -19.00 | b | **0.00154797** |
| V5 Gy / % | 49.68 | 39.39 | -20.71 | b | **0.001435352** |
| V10 Gy / % | 20.98 | 17.54 | -16.40 | b | 0.052687552 |
| V20 Gy / % | 4.29 | 3.08 | -28.21 | b | 0.151956186 |
| V30 Gy / % | 1.53 | 0.7 | -54.25 | b | 0.179712495 |
| V40 Gy / % | 0.46 | 0.06 | -86.96 | b | 0.179712495 |
| Dmean / Gy | 6.57 | 5.59 | -14.92 | b | **0.00438754** |
| *Right coronary artery* |  |  |  |  |  |
| D2% / Gy | 7.17 | 6.87 | -4.18 | b | 0.845056152 |
| V5 Gy / % | 19.05 | 20.25 | 6.30 | b | 0.497914888 |
| V10 Gy / % | 5.21 | 4.69 | -9.98 | b | 0.514669723 |
| V20 Gy / % | 1.21 | 1.63 | 34.71 | b | 1 |
| V30 Gy / % | 0 | 0 |  |  |  |
| V40 Gy / % | 0 | 0 |  |  |  |
| Dmean / Gy | 3.4 | 3.27 | -3.82 | b | 0.270850908 |
| *Posterior descending artery* |  |  |  |  |  |
| D2% / Gy | 2.2 | 2.08 | -5.45 | b | **0.044224421** |
| V5 Gy / % | 5.32 | 7.39 | 38.91 | b | 0.465208819 |
| V10 Gy / % | 0 | 0.58 |  | b | 0.317310508 |
| V20 Gy / % | 0 | 0 |  |  |  |
| V30 Gy / % | 0 | 0 |  |  |  |
| V40 Gy / % | 0 | 0 |  |  |  |
| Dmean / Gy | 1.63 | 1.6 | -1.84 | b | **0.026056039** |

Supplementary Table 2: Dosimetric outcomes of planning in deep inspiration breath-hold (DIBH) and free breathing (FB) for the patients of the complete cohort.

| **LEFT (n = 17)** | **FB** | **DIBH** | **Mean relative  difference (%)** | **Test** | **p-value** |
| --- | --- | --- | --- | --- | --- |
| *Lung total* |  |  |  |  |  |
| Dmean (Gy) | 14.04 | 12.86 | -8.40 | a | **0.001714318** |
| V20Gy (%) | 25.72 | 23.72 | -7.78 | a | **0.031620242** |
| V40Gy / % | 7.6 | 6.31 | -16.97 | a | **2.47E-03** |
| *Lung ipsilateral* |  |  |  |  |  |
| Dmean / Gy | 20.2 | 17.94 | -11.19 | a | **2.61E-04** |
| V5Gy / % | 70.38 | 65.35 | -7.15 | a | **6.26E-05** |
| V10Gy / % | 60.21 | 55.38 | -8.02 | a | **4.08E-04** |
| V20Gy / % | 43.61 | 39.11 | -10.32 | a | **4.19E-03** |
| V40Gy / % | 16.56 | 13.23 | -20.11 | a | **3.33E-04** |
| *Lung contralateral* |  |  |  |  |  |
| Dmean / Gy | 9.88 | 9.3 | -5.87 | a | **0.042332474** |
| V5Gy / % | 58.89 | 54.6 | -7.28 | a | **0.006134563** |
| V10Gy / % | 40.23 | 37.62 | -6.49 | a | 0.059191333 |
| V20Gy / % | 13.45 | 12.83 | -4.61 | a | 0.425869854 |
| V40Gy / % | 1.49 | 1.56 | 4.70 | b | 0.530284597 |
| *Heart* |  |  |  |  |  |
| D2% / Gy | 38.68 | 37.18 | -3.88 | b | 0.127030095 |
| V20 Gy / % | 8.89 | 8.43 | -5.17 | a | 0.54134466 |
| V45 Gy / % | 2.26 | 2.01 | -11.06 | b | 0.157708987 |
| V60 Gy/ % | 0.3 | 0.3 | 0.00 | b | 0.811892227 |
| Dmean / Gy | 7.35 | 7.25 | -1.36 | a | 0.703084138 |
| *Left ventricle* |  |  |  |  |  |
| D2% / Gy | 17.62 | 14.54 | -17.48 | b | 0.055719324 |
| V10 Gy / % | 18.93 | 15.85 | -16.27 | b | 0.332879755 |
| V20 Gy / % | 4.73 | 3.35 | -29.18 | b | 0.213122469 |
| V30 Gy / % | 1.43 | 0.96 | -32.87 | b | 0.32698935 |
| V40 Gy / % | 0.57 | 0.36 | -36.84 | b | 0.345231072 |
| Dmean / Gy | 5.67 | 5.11 | -9.88 | b | 0.124772736 |
| *Right ventricle* |  |  |  |  |  |
| D2% / Gy | 6.82 | 6.88 | 0.88 | a | 0.877284229 |
| V10 Gy / % | 1.91 | 1.9 | -0.52 | b | 0.475245832 |
| V20 Gy / % | 0 | 0 |  |  |  |
| V30 Gy / % | 0 | 0 |  |  |  |
| V40 Gy / % | 0 | 0 |  |  |  |
| Dmean / Gy | 2.82 | 2.87 | 1.77 | a | 0.743237702 |
| *Left atrium* |  |  |  |  |  |
| D2% / Gy | 44.27 | 43.63 | -1.45 | b | 0.352874756 |
| V10 Gy / % | 50.48 | 51.69 | 2.40 | b | 0.325870024 |
| V20 Gy / % | 30.43 | 30.62 | 0.62 | a | 0.934782077 |
| V30 Gy / % | 19.41 | 19.78 | 1.91 | a | 0.838995175 |
| V40 Gy / % | 12.86 | 13.2 | 2.64 | b | 0.861304257 |
| Dmean / Gy | 16.46 | 16.62 | 0.97 | a | 0.84938169 |
| *Right atrium* |  |  |  |  |  |
| D2% / Gy | 15.27 | 15.86 | 3.86 | a | 0.616057924 |
| V10 Gy / % | 9.85 | 10.65 | 8.12 | b | 0.753152365 |
| V20 Gy / % | 1.38 | 2.1 | 52.17 | b | 0.239135782 |
| V30 Gy / % | 0.38 | 0.42 | 10.53 | b | 1 |
| V40 Gy / % | 0.03 | 0.03 | 0.00 | b | 0.712701857 |
| Dmean / Gy | 4.62 | 4.83 | 4.55 | a | 0.397485277 |
| *Left coronary artery* |  |  |  |  |  |
| D2% / Gy | 16.38 | 14.4 | -12.09 | a | 0.147839759 |
| V5 Gy / % | 86.74 | 81.86 | -5.63 | b | 0.179712495 |
| V10 Gy / % | 59.31 | 54.25 | -8.53 | b | 0.284502698 |
| V20 Gy / % | 18.28 | 12.08 | -33.92 | b | 0.463071015 |
| V30 Gy / % | 5.84 | 5.1 | -12.67 | b | 0.317310508 |
| V40 Gy / % | 0 | 0 |  |  |  |
| Dmean / Gy | 12.98 | 11.68 | -10.02 | a | 0.173949769 |
| *Left circumflex coronary* |  |  |  |  |  |
| D2% / Gy | 29.83 | 25.46 | -14.65 | b | **0.004638672** |
| V5 Gy / % | 56.32 | 50.19 | -10.88 | b | **0.009925486** |
| V10 Gy / % | 43.02 | 38.22 | -11.16 | b | 0.099539688 |
| V20 Gy / % | 21.48 | 18.75 | -12.71 | b | 0.332879755 |
| V30 Gy / % | 14.59 | 12.85 | -11.93 | b | 0.498962299 |
| V40 Gy / % | 9.84 | 7.95 | -19.21 | b | 0.128190174 |
| Dmean / Gy | 13.48 | 11.99 | -11.05 | b | **0.007904053** |
| *Left anterior descending* |  |  |  |  |  |
| D2% / Gy | 18.66 | 14.41 | -22.78 | a | **0.015299659** |
| V5 Gy / % | 58.46 | 50.81 | -13.09 | a | **0.017054667** |
| V10 Gy / % | 32.59 | 27.89 | -14.42 | b | 0.148678549 |
| V20 Gy / % | 7.91 | 5.42 | -31.48 | b | 0.085830958 |
| V30 Gy / % | 2.87 | 1.31 | -54.36 | b | 0.179712495 |
| V40 Gy / % | 0.87 | 0.12 | -86.21 | b | 0.179712495 |
| Dmean / Gy | 8.34 | 7.09 | -14.99 | a | **0.042789071** |
| *Right coronary artery* |  |  |  |  |  |
| D2% / Gy | 6 | 6.26 | 4.33 | a | 0.543565372 |
| V5 Gy / % | 18.21 | 19.26 | 5.77 | b | 0.929152765 |
| V10 Gy / % | 2.06 | 2.27 | 10.19 | b | 1 |
| V20 Gy / % | 0 | 0 |  |  |  |
| V30 Gy / % | 0 | 0 |  |  |  |
| V40 Gy / % | 0 | 0 |  |  |  |
| Dmean / Gy | 2.92 | 2.85 | -2.40 | b | 0.690885645 |
| *Posterior descending artery* |  |  |  |  |  |
| D2% / Gy | 2.44 | 2.18 | -10.66 | b | 0.221854908 |
| V5 Gy / % | 7.83 | 8.12 | 3.70 | b | 1 |
| V10 Gy / % | 0 | 0 |  |  |  |
| V20 Gy / % | 0 | 0 |  |  |  |
| V30 Gy / % | 0 | 0 |  |  |  |
| V40 Gy / % | 0 | 0 |  |  |  |
| Dmean / Gy | 1.86 | 1.81 | -2.69 | b | 0.132064599 |

Supplementary Table 3: Dosimetric outcomes of planning in deep inspiration breath-hold (DIBH) and free breathing (FB) for the patients with left lung tumors.

| **RIGHT (n = 15)** | **FB** | **DIBH** | **Mean relative  difference (%)** | **Test** | **p-value** |
| --- | --- | --- | --- | --- | --- |
| *Lung total* |  |  |  |  |  |
| Dmean (Gy) | 15 | 13.44 | -10.40 | a | **0.005285044** |
| V20Gy (%) | 29.05 | 25.11 | -13.56 | a | **0.006200701** |
| V40Gy / % | 9.31 | 7.09 | -23.85 | a | **1.59E-03** |
| *Lung ipsilateral* |  |  |  |  |  |
| Dmean / Gy | 20.75 | 18.3 | -11.81 | a | **7.35E-03** |
| V5Gy / % | 68.43 | 65.17 | -4.76 | a | 7.32E-02 |
| V10Gy / % | 60.53 | 56.25 | -7.07 | a | **3.55E-02** |
| V20Gy / % | 46.07 | 39.8 | -13.61 | a | **4.30E-03** |
| V40Gy / % | 18 | 13.86 | -23.00 | b | **6.10E-04** |
| *Lung contralateral* |  |  |  |  |  |
| Dmean / Gy | 9.32 | 8.84 | -5.15 | a | 0.121498134 |
| V5Gy / % | 57.57 | 54.53 | -5.28 | a | 0.082958541 |
| V10Gy / % | 38.19 | 37.83 | -0.94 | a | 0.814826393 |
| V20Gy / % | 12.43 | 11.08 | -10.86 | b | 0.761535645 |
| V40Gy / % | 0.82 | 0.7 | -14.63 | b | 0.446059549 |
| *Heart* |  |  |  |  |  |
| D2% / Gy | 45.05 | 43.01 | -4.53 | b | 0.055358887 |
| V20 Gy / % | 9.27 | 7.83 | -15.53 | a | 0.116039212 |
| V45 Gy / % | 2.83 | 2.47 | -12.72 | a | **0.008692475** |
| V60 Gy/ % | 0.51 | 0.47 | -7.84 | b | 0.575636914 |
| Dmean / Gy | 7.17 | 6.53 | -8.93 | a | **0.034038065** |
| *Left ventricle* |  |  |  |  |  |
| D2% / Gy | 6.44 | 5.98 | -7.14 | a | 0.271960361 |
| V10 Gy / % | 1.33 | 1.95 | 46.62 | b | 0.400236144 |
| V20 Gy / % | 0 | 0.01 |  | b | 0.317310508 |
| V30 Gy / % | 0 | 0 |  |  |  |
| V40 Gy / % | 0 | 0 |  |  |  |
| Dmean / Gy | 2.42 | 2.23 | -7.85 | b | **0.012451172** |
| *Right ventricle* |  |  |  |  |  |
| D2% / Gy | 8.14 | 6.54 | -19.66 | b | 0.157811369 |
| V10 Gy / % | 5.79 | 3.51 | -39.38 | b | 0.10880943 |
| V20 Gy / % | 0.48 | 0.71 | 47.92 | b | 1 |
| V30 Gy / % | 0 | 0 |  |  |  |
| V40 Gy / % | 0 | 0 |  |  |  |
| Dmean / Gy | 2.96 | 2.49 | -15.88 | b | 0.107758046 |
| *Left atrium* |  |  |  |  |  |
| D2% / Gy | 52.69 | 51.76 | -1.77 | b | 0.638671875 |
| V10 Gy / % | 45.25 | 43.41 | -4.07 | a | 0.528540526 |
| V20 Gy / % | 24.84 | 26.64 | 7.25 | a | 0.438117752 |
| V30 Gy / % | 16.85 | 17.82 | 5.76 | a | 0.548408513 |
| V40 Gy / % | 11.57 | 12.35 | 6.74 | a | 0.505890251 |
| Dmean / Gy | 15.36 | 15.51 | 0.98 | a | 0.86526113 |
| *Right atrium* |  |  |  |  |  |
| D2% / Gy | 39.85 | 32.89 | -17.47 | a | **0.005337672** |
| V10 Gy / % | 28.24 | 25.53 | -9.60 | b | 0.177114041 |
| V20 Gy / % | 16.79 | 12.99 | -22.63 | b | **0.033046944** |
| V30 Gy / % | 9.56 | 6.49 | -32.11 | b | **0.003701749** |
| V40 Gy / % | 4.51 | 2.93 | -35.03 | b | **0.00146885** |
| Dmean / Gy | 10.09 | 8.55 | -15.26 | b | **0.008361816** |
| *Left coronary artery* |  |  |  |  |  |
| D2% / Gy | 13.92 | 11.53 | -17.17 | a | 0.141633743 |
| V5 Gy / % | 88.7 | 86.44 | -2.55 | b | 0.465208819 |
| V10 Gy / % | 42.55 | 38.36 | -9.85 | b | 0.858863013 |
| V20 Gy / % | 6.01 | 5.47 | -8.99 | b | 0.500184257 |
| V30 Gy / % | 0.27 | 0.03 | -88.89 | b | 0.317310508 |
| V40 Gy / % | 0 | 0 |  |  |  |
| Dmean / Gy | 10.11 | 9.18 | -9.20 | a | 0.464578818 |
| *Left circumflex coronary* |  |  |  |  |  |
| D2% / Gy | 11.19 | 12.6 | 12.60 | a | 0.345286999 |
| V5 Gy / % | 41.4 | 36.02 | -13.00 | a | 0.063961121 |
| V10 Gy / % | 13.33 | 14.19 | 6.45 | b | 0.656641934 |
| V20 Gy / % | 0.13 | 3.45 | 2553.85 | b | 0.115851498 |
| V30 Gy / % | 0 | 0 |  |  |  |
| V40 Gy / % | 0 | 0 |  |  |  |
| Dmean / Gy | 5.21 | 5.28 | 1.34 | b | 0.638671875 |
| *Left anterior descending* |  |  |  |  |  |
| D2% / Gy | 11.3 | 9.95 | -11.95 | a | 0.328278434 |
| V5 Gy / % | 39.73 | 26.45 | -33.43 | a | **0.020891279** |
| V10 Gy / % | 7.82 | 5.81 | -25.70 | b | 0.285765219 |
| V20 Gy / % | 0.18 | 0.43 | 138.89 | b | 0.715000655 |
| V30 Gy / % | 0 | 0 |  |  |  |
| V40 Gy / % | 0 | 0 |  |  |  |
| Dmean / Gy | 4.57 | 3.89 | -14.88 | a | 0.146398018 |
| *Right coronary artery* |  |  |  |  |  |
| D2% / Gy | 8.49 | 7.56 | -10.95 | b | 0.846923828 |
| V5 Gy / % | 20.01 | 21.37 | 6.80 | b | 0.38627072 |
| V10 Gy / % | 8.77 | 7.44 | -15.17 | b | 0.345231072 |
| V20 Gy / % | 2.59 | 3.47 | 33.98 | b | 1 |
| V30 Gy / % | 0 | 0 |  |  |  |
| V40 Gy / % | 0 | 0 |  |  |  |
| Dmean / Gy | 3.94 | 3.73 | -5.33 | b | 0.30279541 |
| *Posterior descending artery* |  |  |  |  |  |
| D2% / Gy | 1.93 | 1.97 | 2.07 | b | 0.078054604 |
| V5 Gy / % | 2.48 | 6.57 | 164.92 | b | 0.317310508 |
| V10 Gy / % | 0 | 1.24 |  | b | 0.317310508 |
| V20 Gy / % | 0 | 0 |  |  |  |
| V30 Gy / % | 0 | 0 |  |  |  |
| V40 Gy / % | 0 | 0 |  |  |  |
| Dmean / Gy | 1.36 | 1.36 | 0.00 | b | 0.082200168 |

Supplementary Table 4: Dosimetric outcomes of planning in deep inspiration breath-hold (DIBH) and free breathing (FB) for the patients with right lung tumors.

| **LS 7 positive (n = 22)** | **FB** | **DIBH** | **Mean relative  difference (%)** | **Test** | **p-value** |
| --- | --- | --- | --- | --- | --- |
| *Lung total* |  |  |  |  |  |
| Dmean (Gy) | 16.11 | 14.52 | -9.87 | a | **0.00020377** |
| V20Gy (%) | 31.13 | 27.62 | -11.28 | a | **0.001696284** |
| V40Gy / % | 8.77 | 6.94 | -20.87 | a | **2.98E-04** |
| *Lung ipsilateral* |  |  |  |  |  |
| Dmean / Gy | 21.27 | 18.89 | -11.19 | a | **1.95E-04** |
| V5Gy / % | 73.92 | 69.72 | -5.68 | a | **7.30E-04** |
| V10Gy / % | 64.44 | 60.16 | -6.64 | a | **1.95E-03** |
| V20Gy / % | 46.77 | 41.28 | -11.74 | a | **9.25E-04** |
| V40Gy / % | 17.06 | 13.36 | -21.69 | a | **4.77E-07** |
| *Lung contralateral* |  |  |  |  |  |
| Dmean / Gy | 11.64 | 10.83 | -6.96 | a | **0.003460223** |
| V5Gy / % | 68.14 | 62.86 | -7.75 | a | **0.000397692** |
| V10Gy / % | 49.53 | 46.47 | -6.18 | a | **0.018867291** |
| V20Gy / % | 17.53 | 15.9 | -9.30 | b | 0.236556235 |
| V40Gy / % | 1.61 | 1.57 | -2.48 | b | 0.849795819 |
| *Heart* |  |  |  |  |  |
| D2% / Gy | 48.45 | 46.93 | -3.14 | b | 0.053652394 |
| V20 Gy / % | 10.13 | 9.29 | -8.29 | a | 0.193778284 |
| V45 Gy / % | 3.08 | 2.72 | -11.69 | b | **0.01163652** |
| V60 Gy/ % | 0.53 | 0.51 | -3.77 | b | 0.647236743 |
| Dmean / Gy | 8.02 | 7.69 | -4.11 | a | 0.140922252 |
| *Left ventricle* |  |  |  |  |  |
| D2% / Gy | 13.51 | 11.01 | -18.50 | b | **0.017245169** |
| V10 Gy / % | 10.34 | 8.82 | -14.70 | b | 0.306526583 |
| V20 Gy / % | 2.62 | 1.53 | -41.60 | b | 0.123025194 |
| V30 Gy / % | 0.85 | 0.26 | -69.41 | b | 0.115851498 |
| V40 Gy / % | 0.31 | 0.03 | -90.32 | b | 0.067889155 |
| Dmean / Gy | 4.42 | 3.94 | -10.86 | b | **0.036495955** |
| *Right ventricle* |  |  |  |  |  |
| D2% / Gy | 7.9 | 7.34 | -7.09 | b | 0.726183891 |
| V10 Gy / % | 4.33 | 2.98 | -31.18 | b | 0.414519853 |
| V20 Gy / % | 0.21 | 0.48 | 128.57 | b | 0.654720846 |
| V30 Gy / % | 0 | 0 |  |  |  |
| V40 Gy / % | 0 | 0 |  |  |  |
| Dmean / Gy | 3.04 | 2.93 | -3.62 | b | 0.489442076 |
| *Left atrium* |  |  |  |  |  |
| D2% / Gy | 55.96 | 56.25 | 0.52 | b | 0.137489319 |
| V10 Gy / % | 55.37 | 55.3 | -0.13 | a | 0.980078445 |
| V20 Gy / % | 33.05 | 33.88 | 2.51 | a | 0.69185051 |
| V30 Gy / % | 22.55 | 23.25 | 3.10 | a | 0.655909616 |
| V40 Gy / % | 15.54 | 16.24 | 4.50 | a | 0.559713698 |
| Dmean / Gy | 18.6 | 18.78 | 0.97 | a | 0.822724616 |
| *Right atrium* |  |  |  |  |  |
| D2% / Gy | 29.91 | 27.04 | -9.60 | b | 0.05007267 |
| V10 Gy / % | 20.05 | 20.35 | 1.50 | b | 0.77452898 |
| V20 Gy / % | 8.58 | 8 | -6.76 | b | 0.687373616 |
| V30 Gy / % | 4.38 | 3.6 | -17.81 | b | 0.055179277 |
| V40 Gy / % | 2.22 | 1.54 | -30.63 | b | **0.003494192** |
| Dmean / Gy | 7.7 | 7.35 | -4.55 | b | 0.176191807 |
| *Left coronary artery* |  |  |  |  |  |
| D2% / Gy | 17.09 | 14.62 | -14.45 | b | 0.112871647 |
| V5 Gy / % | 95.68 | 97.33 | 1.72 | b | 0.715000655 |
| V10 Gy / % | 61.03 | 53.84 | -11.78 | b | 0.349101945 |
| V20 Gy / % | 16.64 | 10 | -39.90 | b | 0.260392944 |
| V30 Gy / % | 4.7 | 3.96 | -15.74 | b | 0.179712495 |
| V40 Gy / % | 0 | 0 |  |  |  |
| Dmean / Gy | 13.07 | 11.7 | -10.48 | b | 0.1465168 |
| *Left circumflex coronary* |  |  |  |  |  |
| D2% / Gy | 23.72 | 22.77 | -4.01 | b | 0.424488545 |
| V5 Gy / % | 56.94 | 51.21 | -10.06 | b | **0.02490692** |
| V10 Gy / % | 33.24 | 30 | -9.75 | b | 0.414040003 |
| V20 Gy / % | 11.49 | 12.71 | 10.62 | b | 0.826048864 |
| V30 Gy / % | 8.08 | 7.45 | -7.80 | b | 0.345231072 |
| V40 Gy / % | 5.76 | 4.47 | -22.40 | b | 0.138010738 |
| Dmean / Gy | 10.51 | 9.75 | -7.23 | b | **0.03587532** |
| *Left anterior descending* |  |  |  |  |  |
| D2% / Gy | 16.3 | 13.67 | -16.13 | b | **0.007443428** |
| V5 Gy / % | 54.3 | 44.05 | -18.88 | a | **0.005592619** |
| V10 Gy / % | 23.5 | 19.33 | -17.74 | b | 0.054484128 |
| V20 Gy / % | 5.22 | 3.54 | -32.18 | b | 0.138640634 |
| V30 Gy / % | 2.03 | 1.01 | -50.25 | b | 0.317310508 |
| V40 Gy / % | 0.6 | 0.09 | -85.00 | b | 0.317310508 |
| Dmean / Gy | 7.14 | 6.09 | -14.71 | b | **0.015698997** |
| *Right coronary artery* |  |  |  |  |  |
| D2% / Gy | 7.56 | 7.32 | -3.17 | b | 0.923965931 |
| V5 Gy / % | 22.55 | 23.99 | 6.39 | b | 0.569494278 |
| V10 Gy / % | 5.53 | 5.46 | -1.27 | b | 0.916511908 |
| V20 Gy / % | 0.51 | 2.37 | 364.71 | b | 0.654720846 |
| V30 Gy / % | 0 | 0 |  |  |  |
| V40 Gy / % | 0 | 0 |  |  |  |
| Dmean / Gy | 3.67 | 6.71 | 82.83 | b | 0.482591152 |
| *Posterior descending artery* |  |  |  |  |  |
| D2% / Gy | 2.6 | 2.46 | -5.38 | b | 0.054245472 |
| V5 Gy / % | 7.74 | 10.75 | 38.89 | b | 0.465208819 |
| V10 Gy / % | 0 | 0.85 |  | b | 0.317310508 |
| V20 Gy / % | 0 | 0 |  |  |  |
| V30 Gy / % | 0 | 0 |  |  |  |
| V40 Gy / % | 0 | 0 |  |  |  |
| Dmean / Gy | 1.87 | 1.87 | 0.00 | b | **0.039833215** |

Supplementary Table 5: Dosimetric outcomes of planning in deep inspiration breath-hold (DIBH) and free breathing (FB) for the patients with involvement of lymph node station 7.

| **LS 7 negative (n = 10)** | **FB** | **DIBH** | **Mean relative  difference (%)** | **Test** | **p-value** |
| --- | --- | --- | --- | --- | --- |
| *Lung total* |  |  |  |  |  |
| Dmean (Gy) | 10.93 | 10.08 | -7.78 | a | 0.055415081 |
| V20Gy (%) | 18.81 | 17.22 | -8.45 | a | 0.11702495 |
| V40Gy / % | 7.59 | 6.09 | -19.76 | a | **2.23E-02** |
| *Lung ipsilateral* |  |  |  |  |  |
| Dmean / Gy | 18.66 | 16.38 | -12.22 | a | **2.38E-02** |
| V5Gy / % | 59.68 | 55.45 | -7.09 | a | 5.78E-02 |
| V10Gy / % | 51.39 | 46.16 | -10.18 | a | **2.71E-02** |
| V20Gy / % | 40.35 | 35.38 | -12.32 | a | **2.21E-02** |
| V40Gy / % | 17.63 | 13.89 | -21.21 | a | **2.08E-02** |
| *Lung contralateral* |  |  |  |  |  |
| Dmean / Gy | 5.17 | 5.25 | 1.55 | a | 0.678309742 |
| V5Gy / % | 36.58 | 36.32 | -0.71 | a | 0.855865983 |
| V10Gy / % | 16.71 | 18.47 | 10.53 | a | 0.189344033 |
| V20Gy / % | 2.69 | 3.44 | 27.88 | b | 0.235725651 |
| V40Gy / % | 0.21 | 0.25 | 19.05 | b | 0.179712495 |
| *Heart* |  |  |  |  |  |
| D2% / Gy | 26.74 | 24.47 | -8.49 | b | 0.16015625 |
| V20 Gy / % | 6.74 | 5.65 | -16.17 | b | 0.176296374 |
| V45 Gy / % | 1.31 | 1.14 | -12.98 | b | 0.345447531 |
| V60 Gy/ % | 0.11 | 0.1 | -9.09 | b | 0.705456986 |
| Dmean / Gy | 5.61 | 5.19 | -7.49 | b | 0.10546875 |
| *Left ventricle* |  |  |  |  |  |
| D2% / Gy | 9.89 | 9.47 | -4.25 | b | 0.431640625 |
| V10 Gy / % | 11.42 | 10.45 | -8.49 | b | 1 |
| V20 Gy / % | 2.27 | 3.34 | 47.14 | b | 0.654720846 |
| V30 Gy / % | 0.56 | 1.06 | 89.29 | b | 0.654720846 |
| V40 Gy / % | 0.28 | 0.53 | 89.29 | b | 0.317310508 |
| Dmean / Gy | 3.54 | 3.37 | -4.80 | b | 0.10546875 |
| *Right ventricle* |  |  |  |  |  |
| D2% / Gy | 6.41 | 5.23 | -18.41 | b | 0.173070921 |
| V10 Gy / % | 2.41 | 1.93 | -19.92 | b | 1 |
| V20 Gy / % | 0.25 | 0 | -100.00 | b | 0.317310508 |
| V30 Gy / % | 0 | 0 |  |  |  |
| V40 Gy / % | 0 | 0 |  |  |  |
| Dmean / Gy | 2.54 | 2.17 | -14.57 | b | 0.26261829 |
| *Left atrium* |  |  |  |  |  |
| D2% / Gy | 31.18 | 28.07 | -9.97 | b | 0.845703125 |
| V10 Gy / % | 31.88 | 31.33 | -1.73 | b | 0.888637861 |
| V20 Gy / % | 16.27 | 17.49 | 7.50 | b | 0.865772375 |
| V30 Gy / % | 8.66 | 9.19 | 6.12 | b | 0.685830435 |
| V40 Gy / % | 5.03 | 5.25 | 4.37 | b | 0.892738401 |
| Dmean / Gy | 10.1 | 10.21 | 1.09 | b | 1 |
| *Right atrium* |  |  |  |  |  |
| D2% / Gy | 19.93 | 16.81 | -15.65 | b | 0.514669723 |
| V10 Gy / % | 14.98 | 11.63 | -22.36 | b | 0.224915884 |
| V20 Gy / % | 8.64 | 5.45 | -36.92 | b | 0.248863875 |
| V30 Gy / % | 5.35 | 2.55 | -52.34 | b | 0.138010738 |
| V40 Gy / % | 1.94 | 1.07 | -44.85 | b | 0.10880943 |
| Dmean / Gy | 6.05 | 4.86 | -19.67 | b | 0.440867035 |
| *Left coronary artery* |  |  |  |  |  |
| D2% / Gy | 11.12 | 9.61 | -13.58 | a | **0.014399122** |
| V5 Gy / % | 70 | 54.7 | -21.86 | b | 0.179712495 |
| V10 Gy / % | 30.39 | 31.31 | 3.03 | b | 0.592980098 |
| V20 Gy / % | 3.48 | 6.75 | 93.97 | b | 0.654720846 |
| V30 Gy / % | 0 | 0 |  |  |  |
| V40 Gy / % | 0 | 0 |  |  |  |
| Dmean / Gy | 8.47 | 7.9 | -6.73 | a | 0.298325803 |
| *Left circumflex coronary* |  |  |  |  |  |
| D2% / Gy | 15.31 | 12.09 | -21.03 | b | 0.085830958 |
| V5 Gy / % | 32.59 | 26.7 | -18.07 | b | **0.027707849** |
| V10 Gy / % | 20.01 | 20.25 | 1.20 | b | 0.892738401 |
| V20 Gy / % | 11.45 | 9.09 | -20.61 | b | 0.654720846 |
| V30 Gy / % | 7.02 | 5.46 | -22.22 | b | 0.654720846 |
| V40 Gy / % | 4.04 | 3.68 | -8.91 | b | 0.654720846 |
| Dmean / Gy | 7.61 | 6.85 | -9.99 | b | 0.16015625 |
| *Left anterior descending* |  |  |  |  |  |
| D2% / Gy | 12.81 | 9.34 | -27.09 | a | 0.234049702 |
| V5 Gy / % | 39.51 | 29.15 | -26.22 | b | 0.128190174 |
| V10 Gy / % | 15.44 | 13.61 | -11.85 | b | 0.685830435 |
| V20 Gy / % | 2.23 | 2.08 | -6.73 | b | 0.715000655 |
| V30 Gy / % | 0.42 | 0 | -100.00 | b | 0.317310508 |
| V40 Gy / % | 0.15 | 0 | -100.00 | b | 0.317310508 |
| Dmean / Gy | 5.33 | 4.5 | -15.57 | b | 0.138640634 |
| *Right coronary artery* |  |  |  |  |  |
| D2% / Gy | 6.29 | 5.88 | -6.52 | b | 0.888637861 |
| V5 Gy / % | 11.35 | 12.01 | 5.81 | b | 0.685830435 |
| V10 Gy / % | 4.49 | 3 | -33.18 | b | 0.285049407 |
| V20 Gy / % | 2.75 | 0 | -100.00 | b | 0.317310508 |
| V30 Gy / % | 0 | 0 |  |  |  |
| V40 Gy / % | 0 | 0 |  |  |  |
| Dmean / Gy | 2.8 | 2.29 | -18.21 | b | 0.575403023 |
| *Posterior descending artery* |  |  |  |  |  |
| D2% / Gy | 1.33 | 1.26 | -5.26 | b | 0.498962299 |
| V5 Gy / % | 0 | 0 |  |  |  |
| V10 Gy / % | 0 | 0 |  |  |  |
| V20 Gy / % | 0 | 0 |  |  |  |
| V30 Gy / % | 0 | 0 |  |  |  |
| V40 Gy / % | 0 | 0 |  |  |  |
| Dmean / Gy | 1.08 | 1 | -7.41 | b | 0.39802472 |

Supplementary Table 6: Dosimetric outcomes of planning in deep inspiration breath-hold (DIBH) and free breathing (FB) for the patients without involvement of lymph node station 7.
